# Natural Compound 2,2′,4′-Trihydroxychalcone Suppresses T Helper 17 Cell Differentiation and Disease Progression by Inhibiting Retinoid-Related Orphan Receptor Gamma T

**DOI:** 10.3390/ijms232314547

**Published:** 2022-11-22

**Authors:** Yana Yang, Wenhui Qi, Yanyan Zhang, Ruining Wang, Mingyue Bao, Mengyuan Tian, Xing Li, Yuan Zhang

**Affiliations:** Key Laboratory of Medicinal Resources and Natural Pharmaceutical Chemistry (Shaanxi Normal University), The Ministry of Education, National Engineering Laboratory for Resource Development of Endangered Crude Drugs in Northwest China, College of Life Sciences, Shaanxi Normal University, Xi’an 710119, China

**Keywords:** chalcone derivant, RORγt, Th17 cell differentiation, experimental autoimmune encephalomyeliti, experimental colitis, skin allograft rejection

## Abstract

Retinoid-related orphan receptor γt (RORγt), a vital transcription factor for the differentiation of the pro-inflammatory Th17 cells, is essential to the inflammatory response and pathological process mediated by Th17 cells. Pharmacological inhibition of the nuclear receptor RORγt provides novel immunomodulators for treating Th17-driven autoimmune diseases and organ transplant rejection. Here, we identified 2,2′,4′-trihydroxychalcone (TDC), a natural chalcone derivant, binds directly to the ligand binding domain (LBD) of RORγt and inhibited its transcriptional activation activity. Using three mice models of Th17-related diseases, it was found that the administration of TDC effectively alleviated the disease development of experimental autoimmune encephalomyelitis (EAE), experimental colitis, and skin allograft rejection. Collectively, these results demonstrated TDC targeting RORγt to suppress Th17 cell polarization, as well as its activity, thus, indicating the potential of this compound in treating of Th17-related autoimmune disorders and organ transplant rejection disorders.

## 1. Introduction

Th17 cells, distinguished by the expression of pro-inflammatory cytokines such as IL-17, IL-22, and IL-21, are an important lineage of CD4^+^ T effector (Teff) cells [1,2]. There is overwhelming evidence that Th17 cells exert an essential function in autoimmune inflammation, for instance experimental autoimmune encephalomyelitis (EAE), rheumatoid arthritis, inflammatory bowel disease (IBD), and acute allograft rejection [3,4,5].

The retinoic acid receptor-related orphan receptor gamma t (RORγt; NR1F3), as a ligand-dependent transcriptional factor of Th17 cell differentiation, belongs to the nuclear hormone receptor superfamily. It is reported that RORγt promoted IL-17A production and Th17 polarization. It also taken part in antitumor immunity, autoimmune diseases as well as transplant rejection [6,7]. Based on structural analysis, RORγt regulated target gene expression and physiological function through interacting with both coactivators and corepressors. Therefore, functional inhibitors play a critical role in disease treatment by targeting RORγt, such as digoxin, SR1001, SR1555, ursolic acid and so on [6,8]. However, some drugs discovered did not apply to clinical research due to the severe adverse reactions and low efficacy [9,10,11]. Hence, our goal is to screen out a safe, effective, and natural drug that targets RORγt to treat Th17-associated inflammatory disease.

In this study, compounds that bind to the active domain of RORγt three-dimensional structure were screened from the ZINC database, and the most promising natural compound 2,2′,4′-trihydroxychalcone (TDC), a natural chalcone derivant, was obtained. Chalcone derivant, as the biogenetic precursors of all known flavonoids, is consist of an uncomplicated chemical framework, which can be broadly identified in vegetables and fruits [12,13,14]. Recently, more and more findings concerning to TDC application in antioxidant, anti-inflammatory, anti-tumor activity, and improving memory impairment in various animal models [12,15]. Hence, TDC itself might be potentially used as a lead compound by targeting RORγt for further research on autoimmune diseases as well as organ transplant rejection.

Here, we focused on the positive benefits of TDC, which exhibited significant therapeutic effects on EAE, experimental colitis, and skin allograft rejection. In addition, we demonstrated that TDC, as a prospective and novel pharmacological inhibitor of RORγt, provides a candidate for clinical immunomodulator for treating Th17-related autoimmune disorders and organ transplant rejection disorders.

## 2. Results

### 2.1. Structure-Based Virtual Screening of Small Molecules Targeting RORγt 

**Figure 1:**
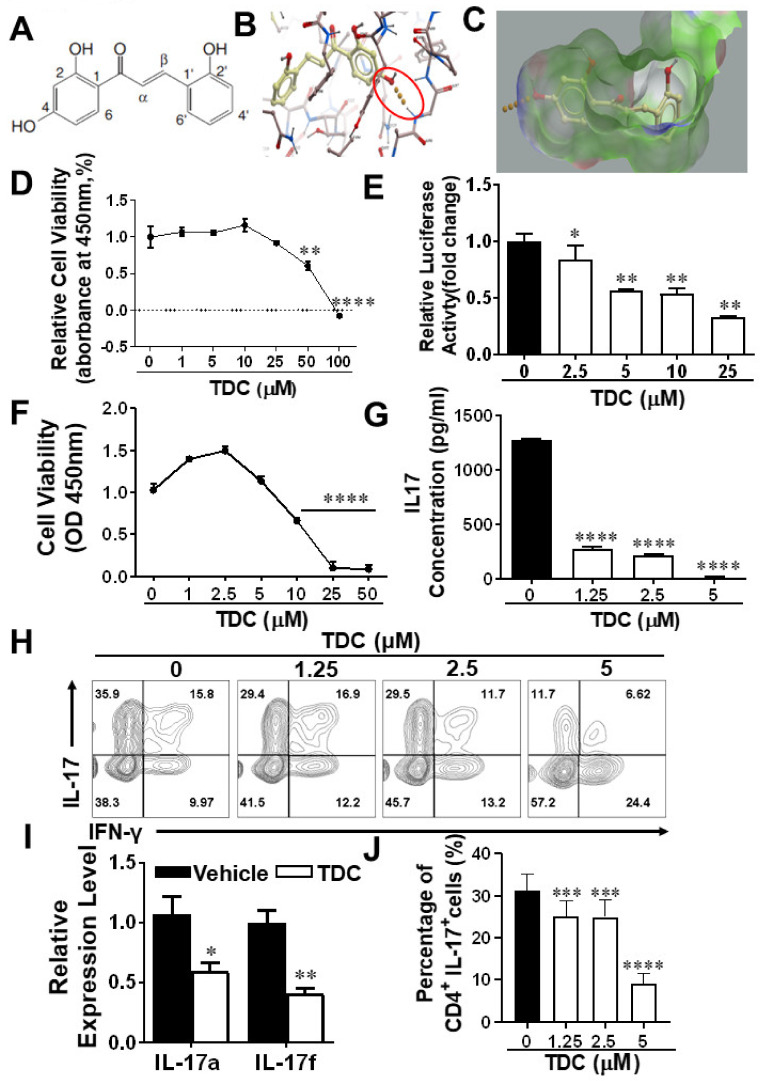
Molecular docking and inhibition of Th17 cell differentiation by TDC targeting RORγt. (**A**) Structural formulas of compound TDC. (**B**) Hydrogen bond connections between TDC and A496 in pocket 1 of RORγt protein (PDB: 5C4T). (**C**) The electron cloud distribution stated TDC binds with V494, F506, W317, I492, and L325 in pocket 1 of 5C4T protein. (**D**) The effect of various concentrations (1–100 μM) of TDC on the viability of HEK293T cells overexpressing mRORγt. (**E**) The impact of TDC on the activity of RORγt luciferase reporter gene. (**F**) The influence of TDC on splenocyte cell viability. (**G**) The level of IL-17 production in Th17 cells after TDC treatment by ELISA. (**I**) qRT-PCR was performed to test *IL-17a* and *IL-17f* genes in TDC and vehicle-treated groups. (**H**,**J**) Intracellular IL-17 staining was used to evaluate the influence of TDC treatment on Th17 cell differentiation by flow cytometry. Data are shown as mean ± SD (*n* = 3 each group) and representative three experiments. * *p* < 0.05, ** *p* < 0.01, *** *p* < 0.001, **** *p* < 0.0001, compared to control group, and determined by one-way ANOVA with Tukey’s multiple comparisons test (**D**–**H**) or unpaired Student’s *t* test (**I**).

In order to screen out novel Retinoid-related orphan receptor γt (RORγt) inhibitors, 60 natural compounds including 2,2′,4′-trihydroxychalcone (TDC) with good binding parameters to RORγt protein were obtained, which have been shown in our previous studies [16] (Figure 1A). The data was showed that TDC formed hydrogen may bond with A496 in RORγt pocket 1. We found that the hydroxybenzene ring of RORγt formed hydrophobic interactions with V494, F506, W317, I492, and L325 in the protein structure. Its *o*-hydroxybenzene ring can also interact with L501, I328, L505, and the surrounding benzene ring to form hydrophobic interactions (Figure 1B,C). 

### 2.2. TDC Suppressed RORγt Transcription Activity In Vitro

We used lentivirus to co-transfer RORγt and CNS2-PIL-17-TK-luciferase reporter genes into 293T cells to establish a dual-luciferase reporter system for the purpose of evaluating the activity of TDC on RORγt transcriptional level. By measuring the viability of 293T, cells were treated with various concentrations (0–100 μM) of TDC, and we chose TDC up to 25 μM for the luciferase reporting experiment (Figure 1D). It can be clearly seen that, in comparison to the vehicle-treated group, TDC markedly suppressed the luciferase activity. At the same time, when the TDC concentration was 25 μM, the expression of luciferase was reduced to 33.63% of the control group (Figure 1E). In summary, these data demonstrated that TDC notably inhibited the transcriptional level of RORγt.

### 2.3. TDC Inhibited Th17 Cell Differentiation In Vitro

In order to optimize the treatment dose of TDC on T cells, the CCK8 activity was measured to detect the effect of different concentrations of TDC on the viability of splenocytes. The results showed that 5 μM TDC was not toxic to splenocytes (Figure 1F). Subsequently, we used different concentrations of TDC (1.25, 2.5, and 5 μM) to perform in vitro experiments to measure the production of Th17-related cytokines. The results showed that TDC significantly inhibited Th17 cell polarization and decreased the secretion of IL-17 in a dose-dependent pattern as against the vehicle group (Figure 1G,H,J). qRT-PCR was used to test the effect of TDC on the gene level of cytokines such as *IL-17a* and *IL-17f*, in which TDC at 2.5 μM significantly inhibited the gene expression of *IL-17a* and *IL-17f* (Figure 1I). Taken together, TDC treatment obviously inhibited Th17 cell differentiation in vitro.

### 2.4. TDC Efficiently Ameliorated the Onset of EAE and Reduced CNS Inflammation 

**Figure 2:**
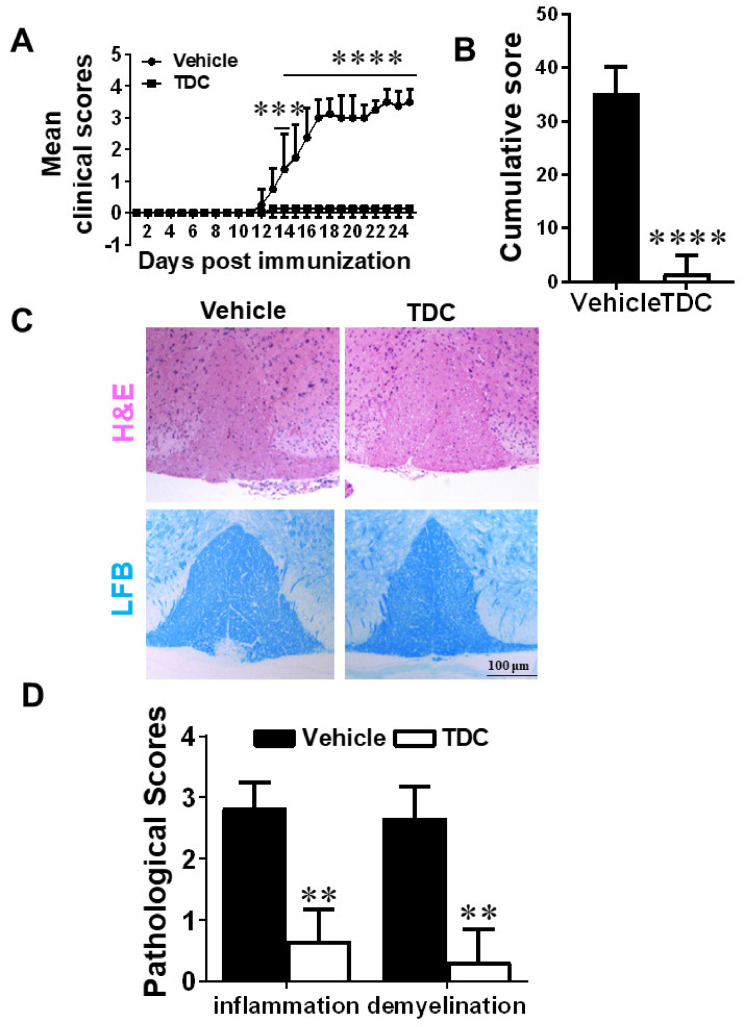
TDC ameliorated the progress of EAE. (**A**) EAE development was evaluated and recorded following a 0–5 scale. (**B**) Cumulative Score of EAE. Clinical scores were cumulated from day 10 p.i. to day 25 p.i. (**C**) Pathological sections at the lumbar level were stained to evaluate the degree of inflammatory infiltration (H&E), (**D**) The pathology scores of inflammation area were evaluated. Data are shown as mean ± SD (*n* = 3 each group) and representative three experiments. ** *p* < 0.01, *** *p* < 0.001, **** *p* < 0.0001, compared to vehicle-treated group, unpaired *t* test (**B**,**D**) or two-way ANOVA with multiple comparisons test (**A**). Scale bar = 100 μm.

In the prophylactic treatment experiment, TDC or vehicle administration started from day 0 p.i. with a dosage of 10 mg/kg/day in EAE mice by intraperitoneal injection. Compared with the vehicle group, TDC administration significantly inhibited clinical scores (Figure 2A) and cumulative clinical scores (Figure 2B) of EAE mice. To further assesses the pathological alterations in the TDC-treated group, histological evaluates of lumbar SCs were performed to determine central nervous system (CNS) inflammation and myelin loss on day 18 p.i. As demonstrated in Figure 2C,D, TDC treatment significantly reduced inflammatory infiltration and demyelination. 

### 2.5. TDC Treatment Blocked the Activation of Debdritic Cells (DCs) and MOG-Reactive T Cells in the CNS

**Figure 3:**
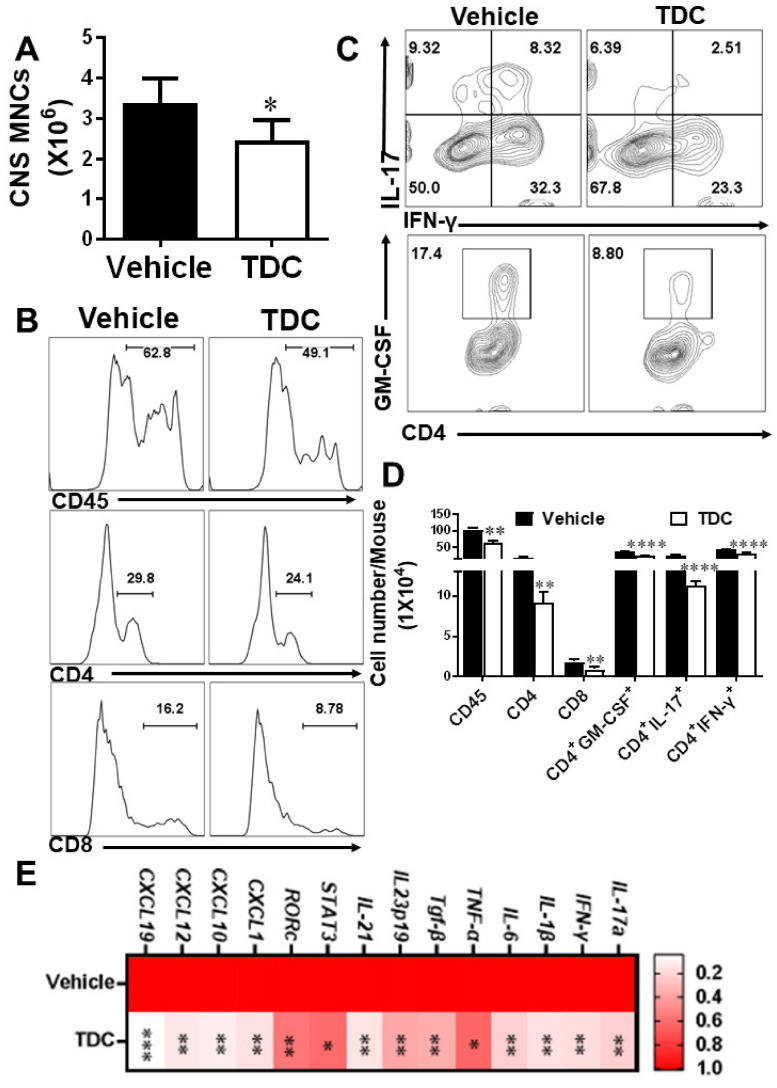
TDC treatment inhibited the inflammatory infiltration of the CNS. (**A**) The number of mononuclear cells in the CNS of EAE mice treated with TDC and vehicle. (**B**) Flow histograms of CD45^+^ cells, CD4^+^ T cells, and CD8^+^ T cells. (**C**) Flow cytometric pseudo-color image of CD4^+^ GM-CSF^+^ cells, Th1, and Th17 cells. (**D**) The statistical results of the number of CD45^+^ cells, CD4^+^ T cells, CD8^+^ T cells, CD4^+^IFN-γ^+^, CD4^+^IL-17^+^, CD4^+^GM-CSF^+^ cells. (**E**) The effect of TDC treatment on the expression of inflammation-related genes in the SC of EAE mice. Data are shown as mean ± SD (*n* = 5 each group) and representative three experiments. * *p* < 0.05, ** *p* < 0.01, *** *p* < 0.001, **** *p* < 0.0001, compared to vehicle-treated group, unpaired *t* test (**A**,**E**) or two-way ANOVA with multiple comparisons test (**D**).

For the purpose of evaluate the impact of TDC on infiltrating pro-inflammatory cells, MNCs in the CNS of EAE mice were harvested on day 20 p.i. and added MOG_35-55_ (10 μg/mL). The number of MNCs, CD45^+^, CD4^+^, and CD8^+^ T cells in the CNS of TDC-treated group was markedly reduced than those of the control group (Figure 3A,B,D). At the same time, the number of pathogenic Th1, Th17, and CD4^+^GM-CSF^+^ cells was remarkably reduced compared with the vehicle group (Figure 3C,D). In addition, TDC inhibited the transcriptional level of cytokines such as *IL-17a*, *GM-CSF*, *IL-23*, *IFN-γ*, *IL-1β*, *IL-6*, *TNF-α*, and chemokines such as *CXCL1*, *CXCL19*, *CXCL12* and *CXCL10* of the SCs of EAE mice (Figure 3E). During the onset of the disease, inflammatory T cells invading the CNS interact with APCs, which lead to reactivation of T cells and activation of APCs [17]. Dendritic cells (DCs) and microglia (CD11b^+^CD45hi^+^), which belong to a class of APCs, played a key role in antigen presentation in innate and adaptive immune mechanism [18,19]. The data demonstrated that the proportion of activated microglia did not change obviously (Supplementary Figure S1A,C). The number of DC cells (CD11c^+^CD80^+^ and CD11c^+^CD86^+^) (Supplementary Figure S1B,D) in the TDC group was less than in the vehicle group, even if there was no significant difference. The similar outcome could be noted in the peripheral tissues (Supplementary Figure S2). 

### 2.6. TDC Alleviated DSS-Induced Colitis 

**Figure 4:**
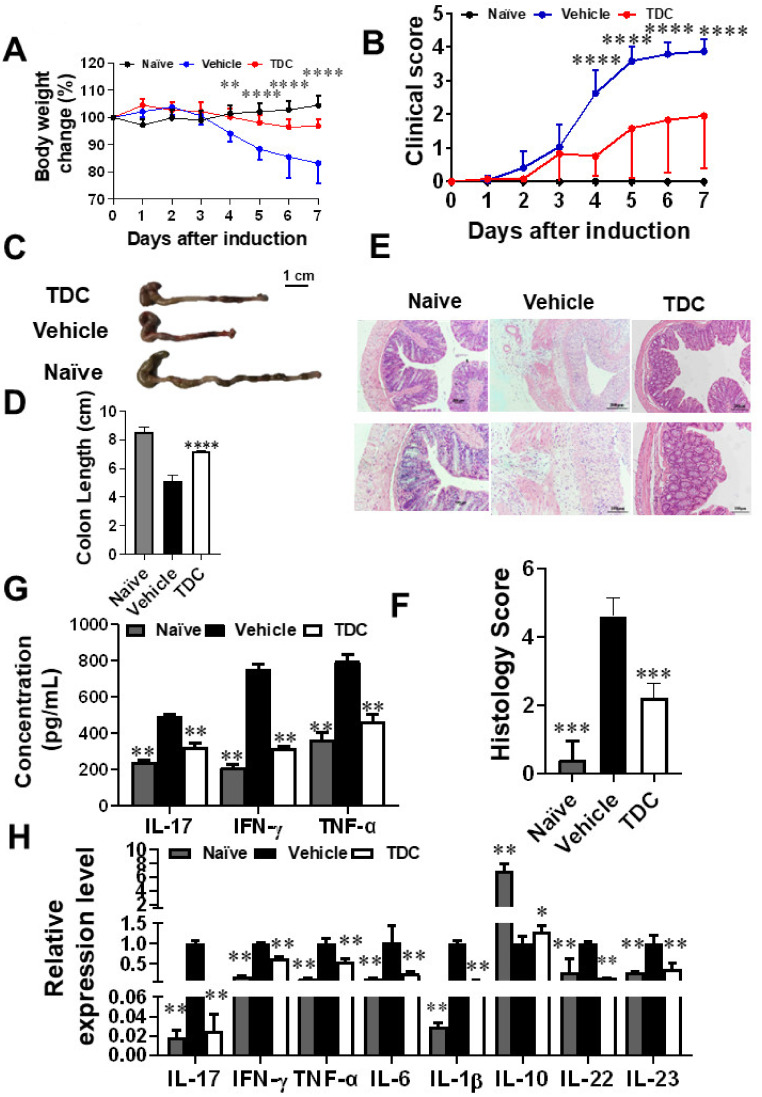
DSS-induced colitis was relieved by TDC. (**A**) The mouse body weight was measured every day during the experiment. (**B**) Clinical score of colitis. (**C**,**D**) Colon length of mice (*n* = 6 each group), scale bar = 1 cm. (**E**,**F**) H&E staining of colonic tissues in the three groups, scale bar = 200 μm (upper row) or 100μm (lower row). (**G**) ELISA assay was used to detect the secretion of IL-17, IL-6, and TNF-α in mouse colon (*n* = 6 per group) (**H**) qRT-PCR was carried out to test the proinflammatory genes in the colitis colon tissues of TDC and vehicle-treated groups. Data are shown as mean ± SD (*n* = 3 each group). * *p* < 0.05, ** *p* < 0.01, *** *p* < 0.001, **** *p* < 0.0001, one-way ANOVA with Tukey’s multiple comparisons test (**D**,**F**–**H**), two-way ANOVA with multiple comparisons test (**A**,**B**).

To study whether TDC has a relief effect on colitis, DSS induced mouse colitis model was employed in this study. As shown in Figure 4, TDC treatment greatly alleviated DSS-induced colitis, as proven by the remarkedly relief of weight loss (Figure 4A), and notably ameliorated of colonic shortening (Figure 4C,D). At the same time, DAI score showed a persistent trend with the above data, indicating that DSS-induced colitis was effectively alleviated by TDC (Figure 4B). Subsequently, H&E staining was used to evaluate colonic mucosal lesions. Compared with the naïve group and vehicle group, the TDC group displayed fewer pro-inflammatory cell infiltration, relatively intact colonic architecture, and minor mucosal damage (Figure 4E,F). Taken together, it was obvious that TDC could improve DSS-induced colitis in the animal model.

### 2.7. TDC Treatment Decreased the Secretion of Pro-Inflammatory Cytokines and Preserved the Proportion of Th17/Treg Cells

**Figure 5:**
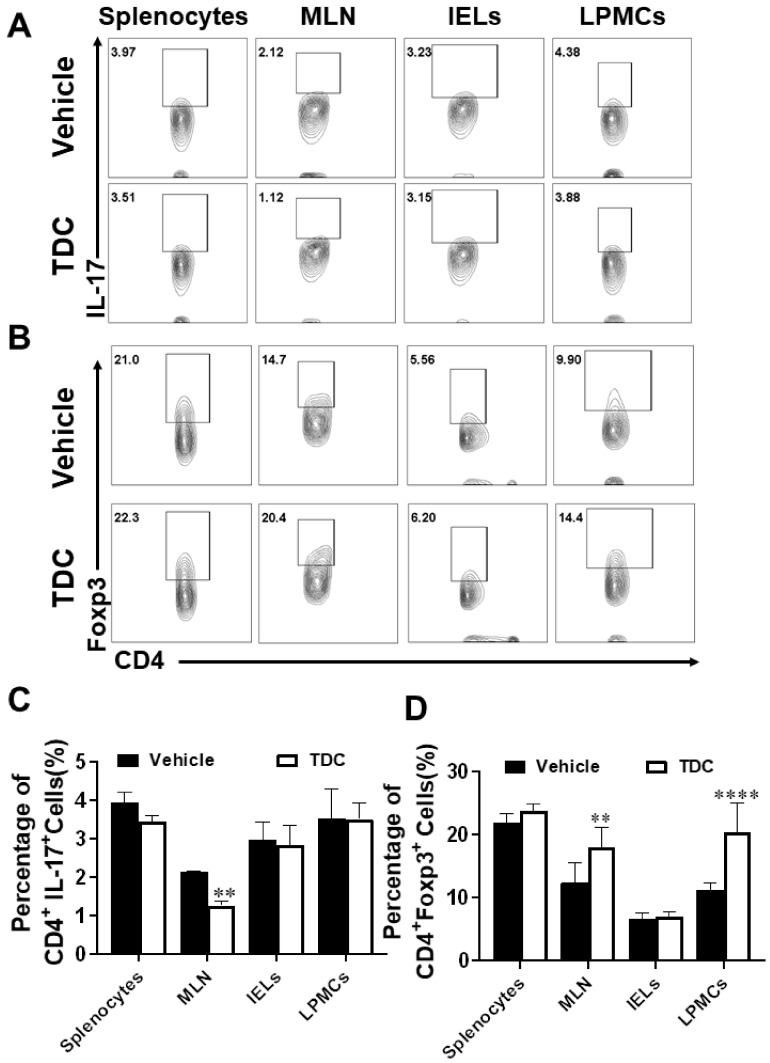
TDC treatment maintained the Th17/Treg balance in DSS-induced colitis mice. (**A**) Flow cytometric pseudo-color images of Th17 cells positive for IL-17 in different organs. (**B**) Flow cytometric pseudo-color images of Treg cells positive for Foxp3 in different organs. (**C**,**D**) The statistical results of the proportion of CD4^+^ IL-17^+^ and CD4^+^ Foxp3^+^ cells. Data are shown as mean ± SD (*n* = 6 each group). ** *p* < 0.01, **** *p* < 0.0001, two-way ANOVA with multiple comparisons test.

To further investigate the function of TDC on T cells in DSS-induced colitis mice, tissues and cells of colitis mice at 7 days after TDC administration were collected. Flow cytometry data displayed that in comparison to the vehicle group, the proportion of Th17 cells in different types in the TDC group was decreased (Figure 5A,C), and the proportion of Treg cells was increased (Figure 5B,D). We used ELISA to check the production of the pro-inflammatory cytokines in colonic tissues. As shown in Figure 4G, TDC treatment significantly inhibited the release of inflammatory factors such as IL-17, IFN-γ, and TNF-α. Next, qRT-PCR was used to detect the expression of inflammation-related genes in the colon, which TDC inhibited the expression of *IL-17*, *IFN-γ*, *TNF-α*, *IL-1β*, *IL-6*, *IL-22*, and *IL-23* genes in the mouse colon and promoted the expression of *IL-10* gene (Figure 4H). In other words, TDC treatment blocked the production of pro-inflammatory cytokines and maintained the balance of Th17/Treg cells to improve colitis in mice.

### 2.8. TDC Alleviated the Transplantation Rejection Responses of Skin Graft In Vivo 

**Figure 6:**
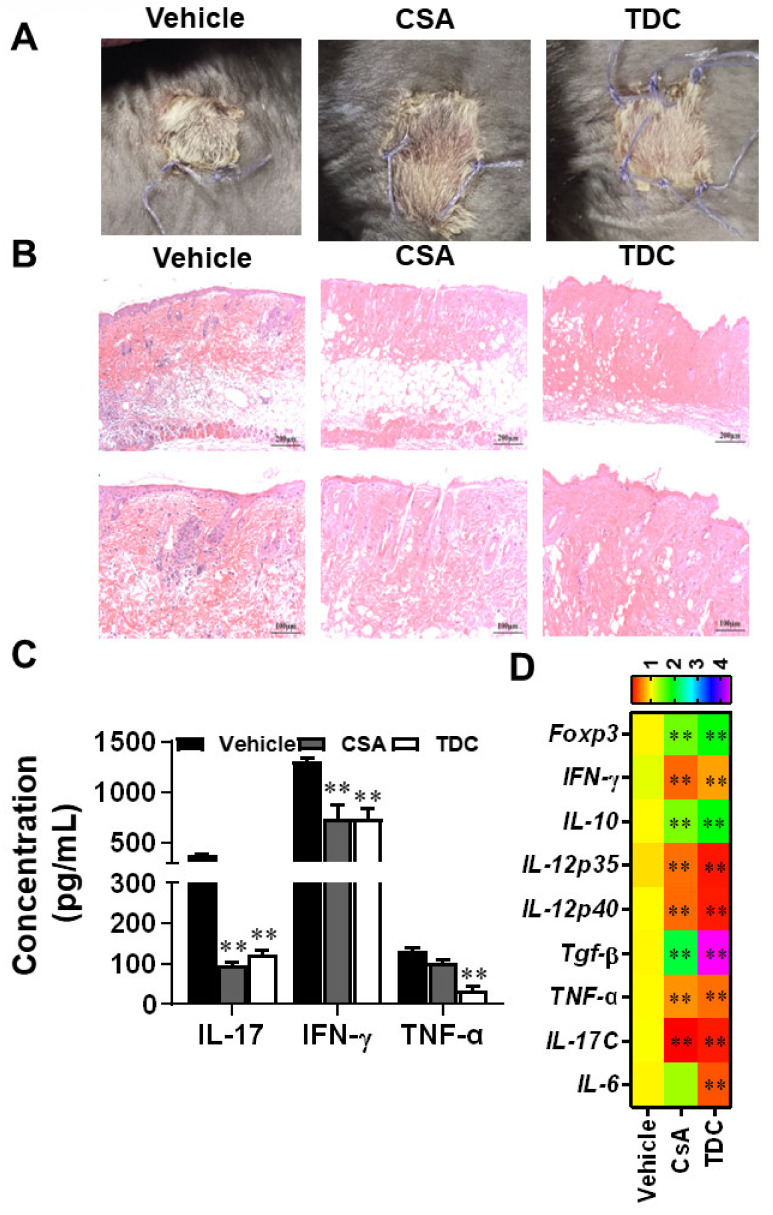
TDC treatment prevented skin allograft rejection. (**A**) The macroscopic aspect of the skin graft 7 days after the skin graft. (**B**) H&E staining of grafted skin slices 7 days after skin transplantation. Scale bar = 200 μm (upper row) or 100 μm (lower row). (**C**) The production of IL-17, IFN-γ and TNF-α in the supernatant of splenocyte culture was detected by ELISA. (**D**) qRT-PCR was performed to test the pro-inflammatory genes in the skin grafts of mice. Data are shown as mean ± SD (*n* = 3 each group). ** *p* < 0.01, one-way ANOVA with multiple comparisons test (**C**) or unpaired Student’s *t* test (**D**).

After 7 days of skin transplantation, the grafts in the vehicle group exhibited scab and necrosis, indicating significant rejection, while the grafts in the CSA group and TDC group survived (Figure 6A). Recipient mice were sacrificed on day 7 of the experiment, and the transplanted skin tissues were stained with H&E. According to the results, inflammatory infiltration in CSA group and TDC group was remarkedly reduced compared with the vehicle-treated group (Figure 6B). Corresponding tissues were collected to check the level of related inflammation factors. As shown in Figure 6C, production of IL-17, IFN-γ, and TNF-α were significantly inhibited by TDC. qRT-PCR was employed to measure the expression of inflammation-related genes in the graft. TDC treatment inhibited the expression of *IFN-γ*, *IL-12p35*, *IL-12p40*, *TGF-β*, *TNF-α*, *IL-17C*, and *IL-6* genes in skin transplants, and upregulated the expression level of *Foxp3* and *IL-10* genes (Figure 6D). These results indicated that TDC inhibited allograft rejection and could be a potential immunomodulator.

### 2.9. TDC Inhibited Skin Graft Rejection in Allogeneic Mice by Maintaining a Balance between Th17 Cells and Treg Cells

**Figure 7:**
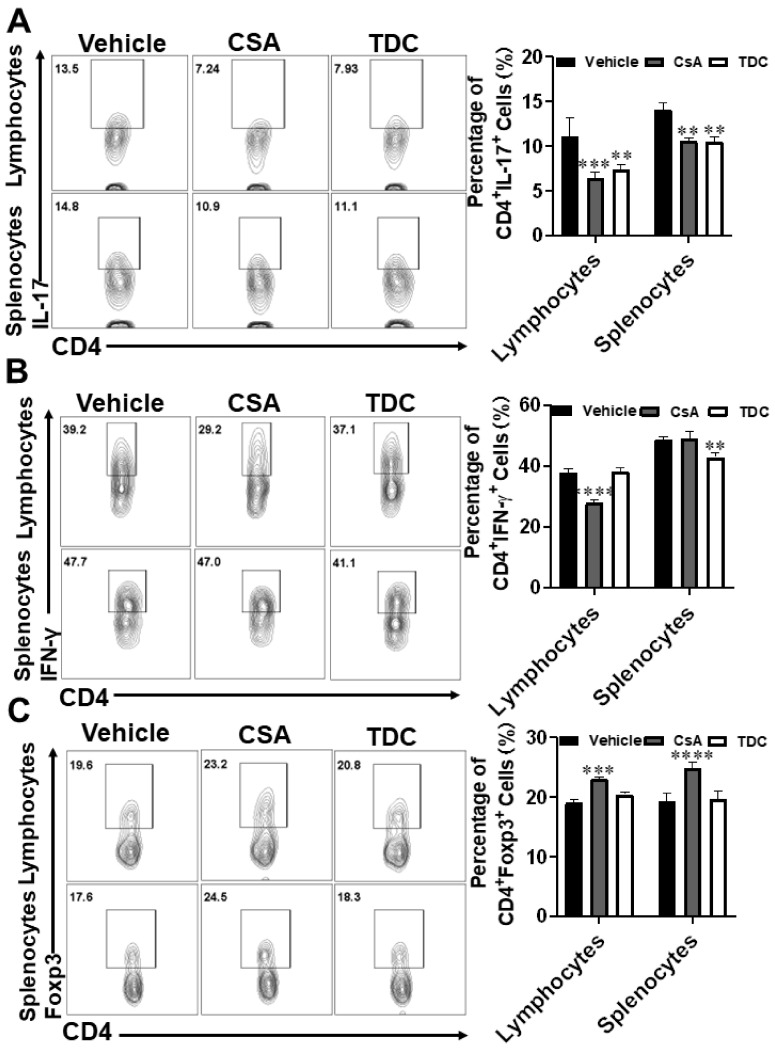
Effects of TDC on Th17, Th1 and Treg cells in splenocytes and lymph nodes of skin transplanted mice. Splenocytes and lymphocytes were prepared in each group at the 8th day after administration. CD4^+^ IL-17^+^, CD4^+^ Foxp3^+^ and CD4^+^ IFN-γ^+^ were detected by flow cytometry. (**A**) Flow cytometric pseudo-color images and statistical results of Th17 cells in different organs. (**B**) Flow cytometric pseudo-color images and statistical results of Th1 cells. (**C**) Flow cytometric pseudo-color images and statistical results of Treg cells in different organs. The results in the panel are expressed as means ± SD and are representative of three experiments (*n* = 6 each group), ** *p* < 0.01, *** *p* < 0.001, **** *p* < 0.0001, two-way ANOVA with multiple comparisons test.

Since Th17 and Treg cells played a critical role in mediating allograft rejection, splenocyte and lymphocyte mononuclear cells of mice were collected for flow cytometry analysis at the 7th day after skin transplantation. In comparison to the vehicle group, the proportion of Th17 cells in the lymphocytes and splenocytes decreased under TDC and CSA treatment (Figure 7A). The proportion of Th1 cells in splenocytes decreased under TDC treatment (Figure 7B). As shown in Figure 7C, the proportion of Treg cells enhanced under CSA and TDC treatment, although there was no significant difference in TDC group. In comparison to the vehicle group, the ratio of CD4^+^ TNF-α^+^ cells decreased, and there was no significant difference in the proportion of IL-4 and IL-10 cells in the CSA and TDC groups (Supplementary Figure S3A,B). These results indicated that TDC played a critical role in suppressing allograft rejection.

## 3. Discussion

This study identified small molecule compounds for the treatment of Th17-driven autoimmune and transplant rejection disorders by high-throughput virtual screening for high-efficiency RORγt inhibitor. Among the 60 RORγt-targeting compounds found by virtual screening, TDC, as a flavonoid extracted from *Glycyrrhiza glabra*, had attracted our attention due to its extensive sources and high safety. We found that TDC significantly inhibited the clinical symptoms of MOG-induced EAE, and additionally effectively alleviated the symptoms of colitis mice and relieved skin graft rejection. Mechanically, TDC regulated Th17/Treg balance to alleviate the disease.

RORγt played a vital role in driving Th17 cell differentiation and IL-17 production, which indicated in the pathology of multiple autoimmune and inflammatory diseases [7,20,21]. However, currently reported drugs targeting RORγt cannot be applied in clinical practice due to the side effects, poor therapeutic action and off-target effect [22]. Our experimental results demonstrated that RORγt-targeting TDC with anti-inflammatory activity had no obvious toxic and side effects on splenocyte viability within the effective dose range. 

CD4^+^ T helper cells regulated immunity and inflammation by differentiating into effector T cell subsets with different functions, such as Th1, Th2, Th17, and Treg [23]. Th1 and Th17, as pro-inflammatory cells, are involved in the pathogenesis of various inflammatory and autoimmune disorders [24,25,26]. However, previous research suggested that IL-23, as the main pro-inflammatory factor and associated with the stable differentiation of Th17, seems to be necessary for maintaining chronic inflammation [27]. The inhibitor targeting RORγt may lessen not only the Th17 reaction but also influence the proportion of Tregs [27]. The studies have also shown that there is a balance between the expression level of RORγt and Foxp3, which regulates the balance of Treg and Th17 [28]. In addition, Treg inhibits IL-12 production by producing IL-10 and TGF-β, thereby inhibiting IL-12-induced Th1 cell differentiation. Based on these data, we hypothesized that TDC inhibits Th17 and Th1 differentiation and maintains the balance between T cells and Treg cells by acting as an inhibitor of RORγt.

We found that TDC could alleviate the pathology of EAE disease through reduced Th1 and Th17 in the CNS (Figure 3). Th17 cells not only exhibit a critical function in multiple sclerosis but also in IBD [29]. However, it is reported that some drugs for IBD have too many side effects to be used in the clinic, and there is a failure of anti-IL-17 strategies for the treatment of Crohn’s disease [8,30,31,32]. Previous studies have shown that Foxp3^+^ RORγt^+^ Treg cells exist in the intestines of IBD patients and were enhanced by RORγt inhibition [33,34]. We thus chose a colitis animal model which was induced by DSS to assess the function of TDC. In our study, TDC improved DSS-induced colitis, decreased the production of pro-inflammatory cytokines, and maintained the balance of Th17/Treg cells in mice (Figure 4). At last, skin transplantation was used to further confirm the immunomodulatory function of TDC. Similarly, TDC inhibited skin graft rejection in allogeneic mice by maintaining the balance between Th17 cells and Treg cells (Figure 6).

Taking the previous reporters into account, together with our own scientific study, in this work, TDC targeting RORγt was identified through virtual screening and in vitro validation. Furthermore, three animal models of Th17-driven disorders were carried out to evaluate the immunomodulatory potential of TDC in vivo. In terms of mechanism, we found that TDC targeting RORγt can inhibit Th17 cell differentiation and its function. Meanwhile, it could stabilize Foxp3 expression and regulate Th17/Treg balance. The present study suggests that TDC was a prospective immunomodulatory agent for treating autoimmune diseases and transplant rejection which paving the way for a future clinical study.

## 4. Material and Methods

### 4.1. Virtual Screening and Molecular Docking

Structure-based virtual screening plays a critical role in drug exploration. Candidate inhibitors targeting RORγt (PDB: 5C4T) were screened out from the zinc natural products database (http://zinc.docking.org) containing 150,000 natural small molecule compounds using Autodock Vina and PyRx software, which acquired on 4 July 2017 [35]. Receptor models and small-molecule libraries were built, followed by scoring and structural docking of small-molecule compounds that fit the receptor-small-molecule structure. Generally speaking, we believe that the higher the score, the more likely the ligand will bind to the active center of the protein.

### 4.2. Luciferase Reporter Assays

We transfected HEK293T cells with a vector overexpressing of RORγt and expressing an IL-17 promoter-driven luciferase. After cells were treated with TDC (0–25 μM) for 16 h, ONE-Glo™ Luciferase Assay kit (Promega, Madison, WI, USA) was employed to monitor the luciferase activity, which was determined by GloMax [36].

### 4.3. EAE Induction and Treatment

Female C57BL/6 mice (aged 8 weeks) were purchased from Experimental Animal Center, Air Force Medical University (Xi’an, Shaanxi, China). All experimental protocols on mice were carried out following the standardized guidelines and specifications (No. ECES-2015-0247), which were approved and supported by the Institutional Animal Ethics Committee of Shaanxi Normal University. In order to construct the EAE mouse model, mice were immunized at the two points of spinal cord (SC) cervical expansion and lumbar expansion on the back with 200 μg of myelin oligodendrocyte glycoprotein peptide 35-55 (MOG_35-55_) (GenScript, Nanjing, China) in 200 μL of hybrid emulsion containing incomplete Freund’s adjuvant (Sigma-Aldrich, St. Louis, MO, USA) with 5 mg/mL heat-killed *Mycobacterium tuberculosis* H37Ra (BD, Franklin Lakes, NJ, USA) [37]. All animals were intraperitoneally (i.p.) injected with 200 ng pertussis toxin (Sigma-Aldrich) in PBS on days 0 and 2 post-immunization (p.i.). TDC (10 mg/kg/day), dissolved in a solvent consisting of dimethyl sulfoxide (DMSO, 3%), Kolliphor (10%), and 5% glucose solution (87%), was injected i.p. to EAE mice every day. The clinical scores were recorded daily in a double-blind manner, following a 0–5 scale described previously [36].

### 4.4. DSS-Induced Colitis 

Male C57BL/6 mice (aged 8 weeks) were induced colitis by adding 3% dextran sulfate sodium (DSS) to drinking water for a week [38]. Gavage administered these animals with vehicle or TDC (100 mg/kg/day) during the DSS treatment. During DSS treatment, mice were observed and recorded daily for morbidity and body weight. At the same time, we scored the pathological characteristics of each mouse daily, involving stool consistency, presence of blood in the stool, and weight loss. Disease activity index (DAI) was evaluated by a clinical score of colitis mice in a double-blind manner according to the previous study [39].

### 4.5. Skin Grafting

BALB/c (aged 8 weeks old, male) and C57BL/6 mice (aged 8 weeks old, male) were used to construct the skin grafting model. Under sterile conditions, back skin of the BALB/c mouse (10 mm × 10 mm) was transplanted to the back of the C57BL/6 mouse. Firstly, we adjust the skin patch and the recipient skin. Next, we suture with 6-0 thread to make them combined tightly, promoting the growth of the skin. Finally, the recipient mice were covered with a sterile gauze and bandaged [40]. We randomly divided the mice into three groups with different treatment [Vehicle, TDC (10 mg/kg/day), or Cyclosporine A (CSA, 20 mg/kg/day)]. The treatment was performed for 7 days from the day of transplantation. The skin grafts on the back of recipient mice were observed daily for inflammation, edema, necrosis, scab, and shedding. We consider more than 80% of the skin necrosis to be complete rejection.

### 4.6. Histopathological Analysis

Mice in the experimental group were sacrificed at the corresponding time points and perfused with cold phosphate buffered saline (PBS) transcardially. Tissues [brain, spinal cord (SC), distal colon, and grafts] were fixed with 4% paraformaldehyde for 24 h and then sectioned into 5-micron slices. The distal colon and graft skin were stained with hematoxylin-eosin (H&E), and the brain and SC were stained with H&E and Luxol Fast Blue (LFB). Slides were assessed and recorded in a double-blinded fashion to assess inflammatory cell infiltration or demyelination accorded to the previous protocols [38]. 

### 4.7. Th17 Cell Polarization

Splenocytes were isolated and co-incubated with Con A (5 μg/mL) and TDC at different concentrations (1, 2.5, 5, 10, 25, and 50 μM) for 18 h. The cell viability was determined by Cell counting kit-8 (ZETA, Arcadia, CA, USA). An optimized dose with no cytotoxicity was used in the subsequent in vitro experiments. Isolation of CD4^+^ T lymphocytes was carried out following the protocols of the Naïve CD4^+^ T Cell Separation Kit (Miltenyi Biotec, Bergisch Gladbach, Germany), then incubated with 5 μg/mL of anti-CD3, 2 μg/mL of anti-CD28 (Bioxcell, Lebanon, NH, USA), 2 ng/mL of TGF-β, 20 ng/mL of IL-6, 10 ng/mL of IL-1β (Peprotech, Cranbury, NJ, USA), 10 μg/mL of anti-IL-4, and 10 μg/mL of anti-IFN -γ (Bioxcell, Lebanon, NH, USA) to induce Th17 cell differentiation. Three days later, cells were harvested in order to evaluate the percentage of Th17 cells by flow cytometry. Simultaneously, a cell medium was gathered to determine IL-17 production by ELISA. 

### 4.8. Cytokine Measurement by Flow Cytometry and ELISA

Spleenocytes, mesenteric lymph nodes, intestinal intraepithelial lymphocytes (IELs), lamina propria mononuclear cells (LPMCs), and mononuclear cells (MNCs) were isolated, and the process of cell extraction are described previously [36]. These separated cells were seeded in vitro at different time, and stimulated by PMA (50 ng/mL, Sigma-Aldrich, Steinheim, Germany), ionomycin (500 ng/mL), and GolgiPlug (Thermo Fisher Scientific, Waltham, MA, USA) for 5 h. Subsequently, cells were stained on the surface, fixed, and permeated, and then internally stained with mouse Abs (Bioxcell, Lebanon, NH, USA). Flow cytometry analysis was carried out on the CytoFLEX S Flow Cytometer (Beckman coulter, Brea, CA, USA) and the results were evaluated with FlowJo (Treestar, Ashland, Wilmington, DE, USA). The cell supernatants were gathered and cytokine production (IFN-γ, IL-17, GM-CSF, and TNF-α) were determined by ELISA (R&D Systems, Minneapolis, MN, USA).

### 4.9. Real-Time Quantitative PCR 

RNA Preparation Pure Tissue Kit (Tiangen, Beijing, China) was used to isolate total ribonucleic acid from the spinal cords of EAE experimental mice, the colon of colitis mice, and the skin graft of skin transplanted mice. cDNA was obtained through using Prime Script™ RT Master Mix Kit by using reverse transcription PCR, and then added to ChamQ™ SYBR^®^qPCR Master Mix (TaKaRa, Shiga, Japan) for real-time quantitative PCR (qRT-PCR). Data were quantitatively analyzed on the LightCycler^®^ 96 system (Roche, Shanghai, China). Mouse glyceraldehyde 3-phosphate dehydrogenase (*GAPDH*) gene, as a housekeeping gene, was used as a standardization control. 

### 4.10. Statistical Analysis

All data are presented as the mean ± SD, and statistical analyses were performed using GraphPad Prism 6 software (GraphPad, La Jolla, CA, USA). When comparing two groups, data were assessed by unpaired Student’s *t* test. When comparing multiple groups, experimental results were evaluated by one-way ANOVA with Tukey’s multiple comparisons test or two-way ANOVA with multiple comparisons test.

## Data Availability

The data presented in this study are available upon request to corresponding author.

## Supplementary Materials

The following supporting information can be downloaded at: https://www.mdpi.com/article/10.3390/ijms232314547/s1.

## Abbreviations

APCs: Antigen-presenting cells; CSA, Cyclosporine A; CNS, Central nervous system; DCs, Dendritic cells; DAI, Disease activity index; DSS, Dextran sulfate sodium; EAE, Experimental autoimmune encephalomyelitis; H&E, Hematoxylin and eosin; IL-17, Interleukin-17; IBD, Inflammatory bowel disease; IELs, Intraepithelial lymphocytes; i.p., Intraperitoneally; KD, Equilibrium dissociation constant; LFB, Luxol fast blue; LPMCs, Lamina propria mononuclear cells; MST, Microscale thermophoresis; MNCs, Mononuclear cells; MLNs, Mesenteric lymph nodes; MS, Multiple sclerosis; qRT-PCR, Real-time quantitative PCR; RORγt, Retinoic acid receptor-related orphan gamma t; TDC, 2,2′,4′-trihydroxychalcone; Th17, T-helper 17; Th1, T-helper 1; Tregs, Regulatory T cells.
