# Supplementary material for: Natural Compound 2,2′,4′-Trihydroxychalcone Suppresses T Helper 17 Cell Differentiation and Disease Progression by Inhibiting Retinoid-Related Orphan Receptor Gamma T
Source: Int J Mol Sci. 2022 Nov 22;23(23):14547. doi: 10.3390/ijms232314547 (PMC9737070; doi:10.3390/ijms232314547)
Supplement: Supplementary file 1 [file ijms-23-14547-s001.zip › ijms-1978987-supplementary.pdf]

Supplementary Figure 1

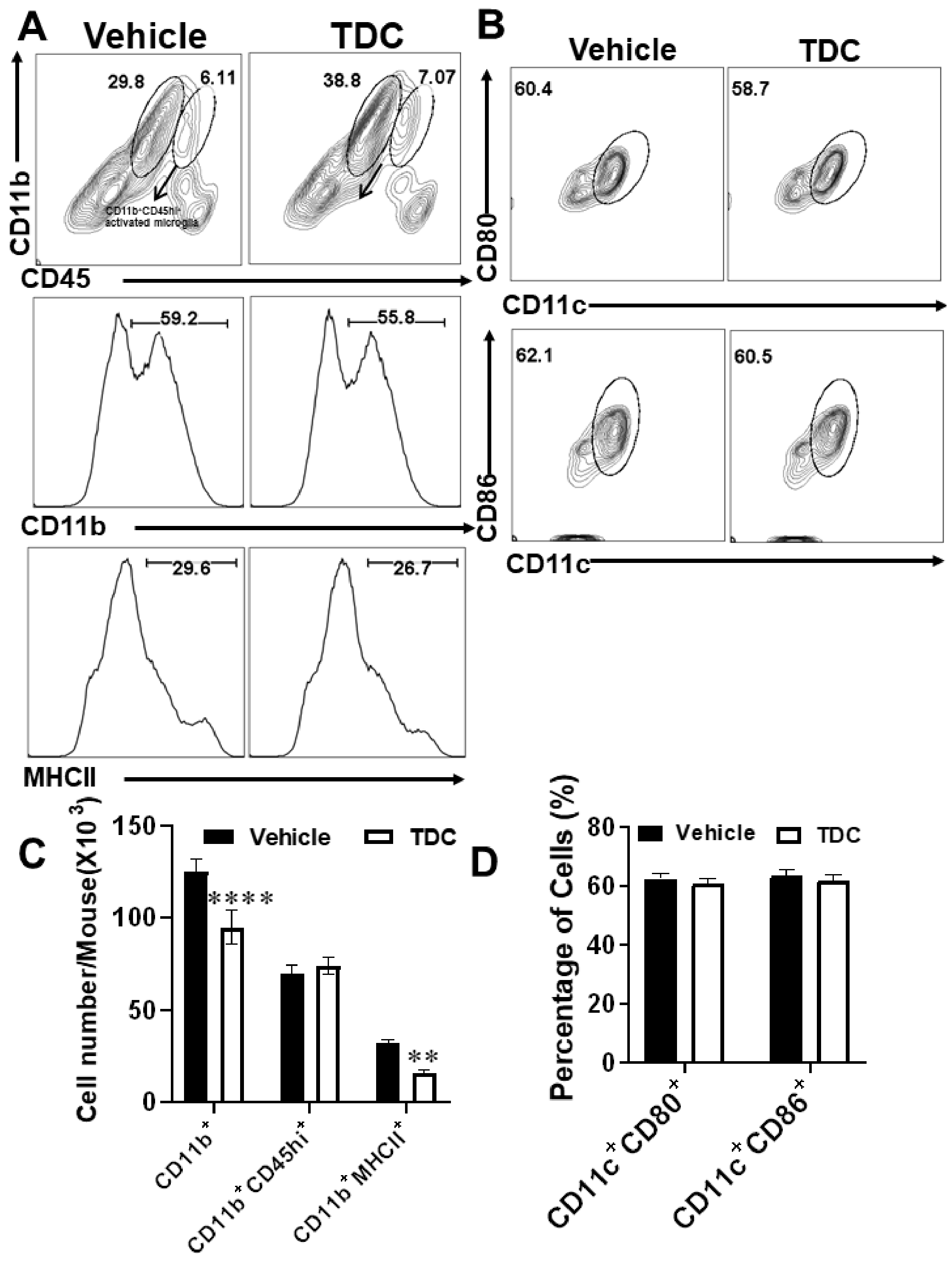

Supplementary Figure S1. The effect of TDC treatment on the activation of microglia and DCs in central nervous system. (A) Flow cytometry analysis of activated microglia (CD11b^{+} CD45^{hi+}) CD11b^{+} cells and CD11b^{+} MHCII^{+} cells. (B)

The proportion of $CD11c^+$ $CD80^+$ and $CD11c^+$ $CD86^+$ cells in $CD45^+$ gate in CNS. (C) The statistical results of the proportion of $CD11b^+CD45hi^+$, $CD11b^+$ and $CD11b^+MHCII^+$ cells. (D) The percentage of $CD11c^+CD80^+$ and $CD11c^+CD86^+$ cells in the CNS was counted. Data are shown as mean ± SD (n = 6 mice per group) and are representative of three experiments. ** $P < 0.01$, **** $P < 0.0001$, two-way ANOVA with multiple comparisons test.

Supplementary Figure 2

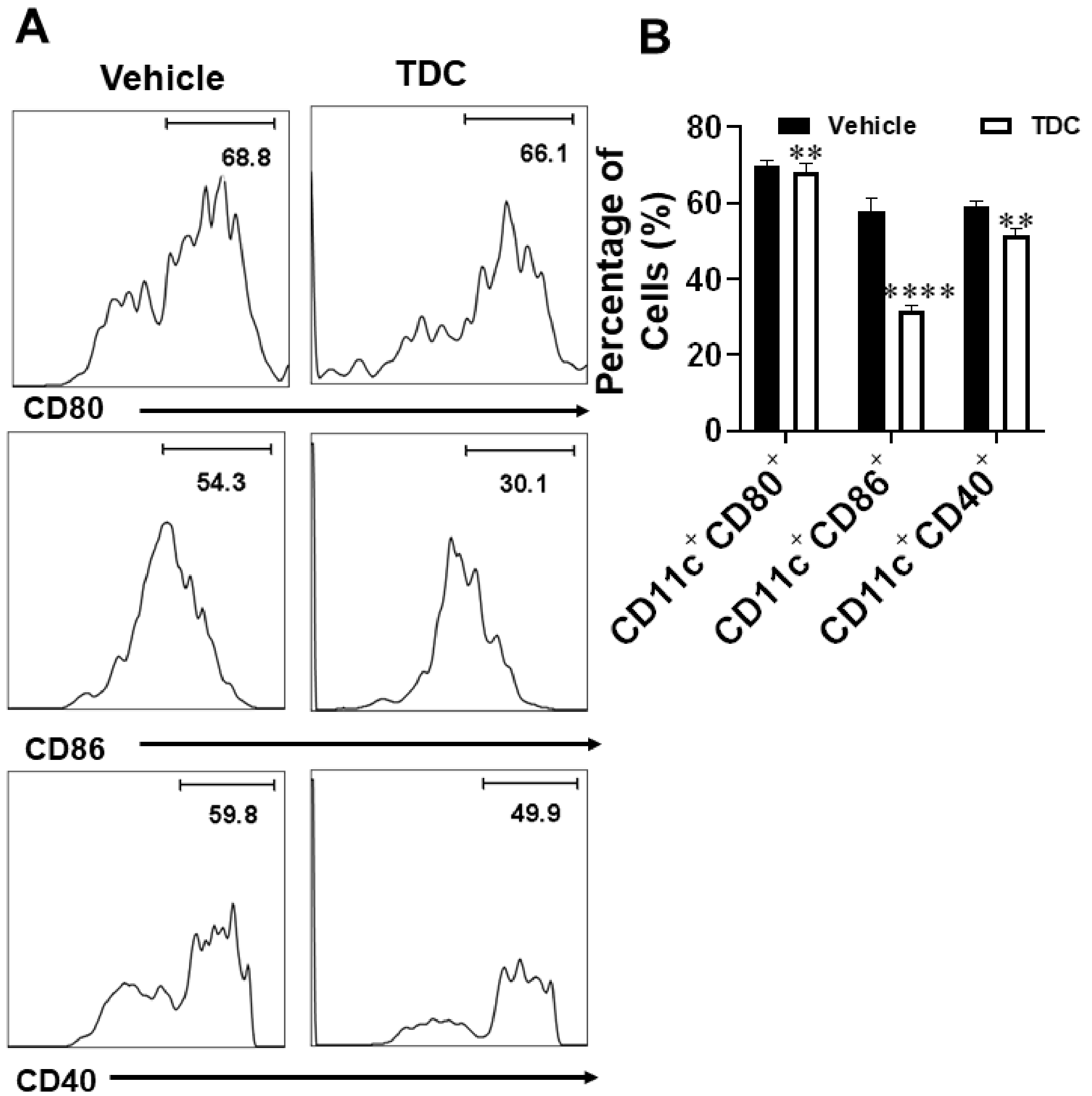

Supplementary Figure S2. The effects of TDC treatment on DCs in periphery. (A) The percentage of CD11c$^+$ CD80$^+$, CD11c$^+$ CD86$^+$ and CD11c$^+$ CD40$^+$ cells in CD45$^+$ gate in peripheral nervous systems. (B) The percentage of CD11c $^+$CD80$^+$,

$CD11c^+CD86^+$, $CD11c^+ CD40^+$ cells was counted. Data are shown as mean ± SD (n = 6 mice per group), ** $P < 0.01$,**** $P < 0.0001$, two-way ANOVA with multiple comparisons test.

Supplementary Figure 3

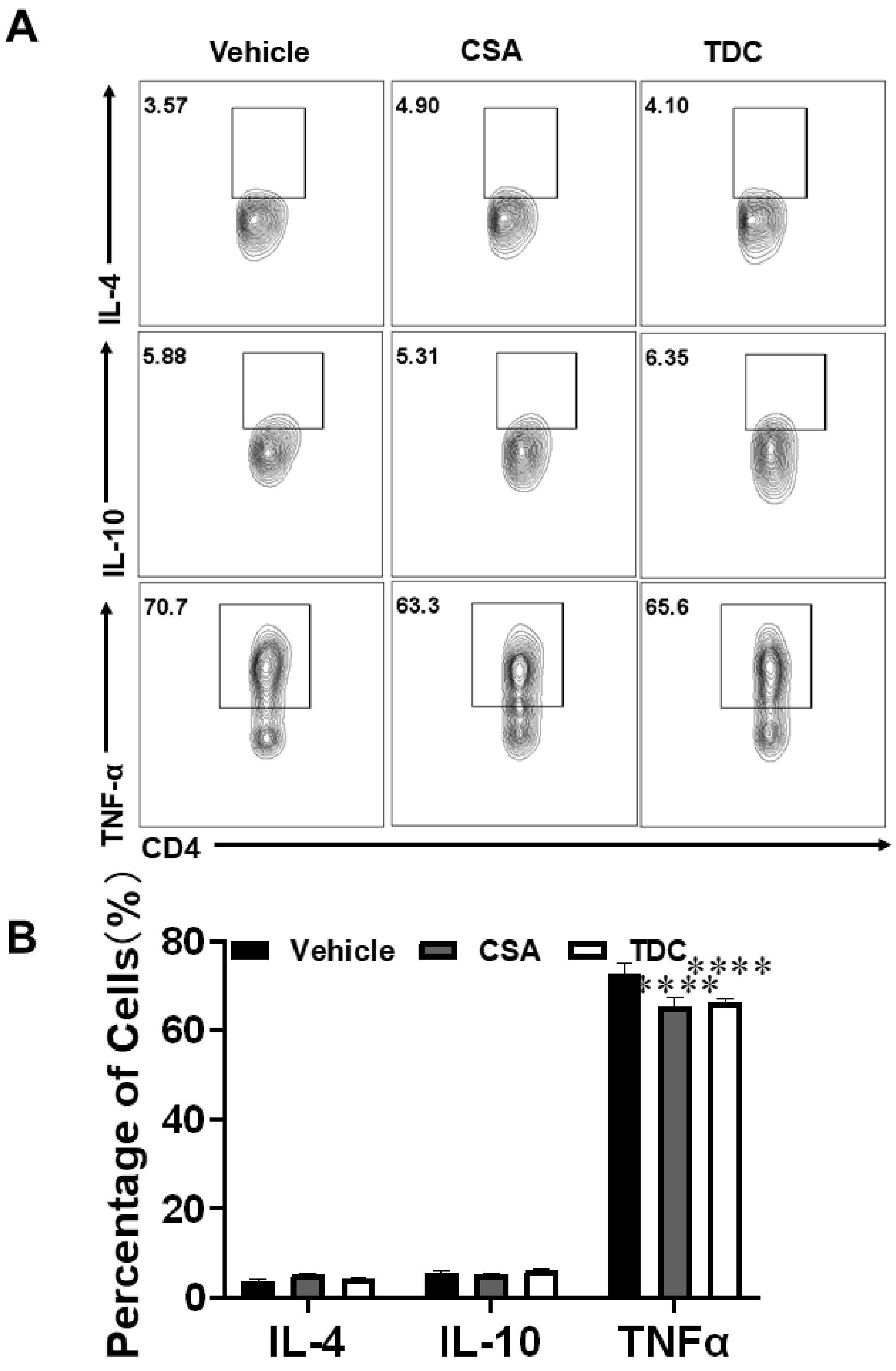

Supplementary Figure S3. Effects of TDC on T cells in splenocytes of skin transplanted mice. (A) Flow cytometric pseudo-color image of $CD4^{+}IL\text{-}4^{+}$, $CD4^{+}IL\text{-}10^{+}$ and $CD4^{+}TNF\text{-}\alpha^{+}$ in splenocyte cells. (B) Statistical comparison of $CD4^{+}IL\text{-}4^{+}$, $CD4^{+}IL\text{-}10^{+}$ and $CD4^{+}TNF\text{-}\alpha^{+}$ in different groups' splenocytes. Data are presented as mean values ± SD and are representative of three experiments (*n* =3 each group) , ****$P < 0.0001$, two-way ANOVA with multiple comparisons test.
